# Dielectrophoretic Crossover Frequency of Single Particles: Quantifying the Effect of Surface Functional Groups and Electrohydrodynamic Flow Drag Force

**DOI:** 10.3390/nano10071364

**Published:** 2020-07-13

**Authors:** Yun-Wei Lu, Chieh Sun, Ying-Chuan Kao, Chia-Ling Hung, Jia-Yang Juang

**Affiliations:** Department of Mechanical Engineering, National Taiwan University, Taipei 10617, Taiwan; r05522502@ntu.edu.tw (Y.-W.L.); r05522516@ntu.edu.tw (C.S.); r07522512@ntu.edu.tw (Y.-C.K.); r06522502@ntu.edu.tw (C.-L.H.)

**Keywords:** dielectrophoresis (DEP), electrohydrodynamics (EHD), electrothermal effect (ETE), AC electro-osmosis (ACEO), electric double layer (EDL), co-ion adsorption, surface conductance, optical tweezers (OTs), surface charge density

## Abstract

We present a comprehensive comparison of dielectrophoretic (DEP) crossover frequency of single particles determined by various experimental methods and theoretical models under the same conditions, and ensure that discrepancy due to uncertain or inconsistent material properties and electrode design can be minimized. Our experiment shows that sulfate- and carboxyl-functionalized particles have higher crossover frequencies than non-functionalized ones, which is attributed to the electric double layer (EDL). To better understand the formation of the EDL, we performed simulations to study the relationship between initial surface charge density, surface ion adsorption, effective surface conductance, and functional groups of both functionalized and nonfunctionalized particles in media with various conductivities. We also conducted detailed simulations to quantify how much error may be introduced if concurrent electrohydrodynamic forces, such as electrothermal and electro-osmotic forces, are not properly avoided during the crossover frequency measurement.

## 1. Introduction

Dielectrophoresis (DEP) is a phenomenon in which suspended dielectric particles are polarized and moved relative to the medium by a non-uniform alternating current (AC) or direct current (DC) electric field [1,2,3]. AC DEP has been widely used in lab-on-a-chip systems [4,5] for particle/cell trapping [6,7,8,9,10,11], manipulation [12,13,14,15], and tissue engineering [16,17,18,19]. The magnitude and direction of the AC DEP force depend on the electric field gradient, the applied frequency, and the polarizability of the particle, which depends on the dielectric properties of the particle and the medium. The crossover frequency, at which the DEP force reverses its direction, is of particular importance since it is a key characteristic that enables particle/cell separations [20,21] and contains critical information on the mechanisms of the electric double layer (EDL) [22]. Despite intensive investigation over the years, accurate determination of the DEP crossover frequency, both theoretically and experimentally, of a single particle remains challenging. Several experimental methods were proposed to measure the crossover frequency of colloidal particles by using observation and estimation [23,24,25,26], the direct optical tweezers (OTs) force method [27,28], and a combination of optical tweezers and phase-sensitive lock-in detection (phase shift method) [28,29]. However, experimental results reported by different groups using different methods still show a certain discrepancy, especially for larger particles and/or a more conductive medium [23,29,30]. A significant discrepancy is also observed between experimental results and the theory based on Clausius–Mossotti (CM) factor, especially for larger particles, e.g., 5 μm, in the low-frequency regime [27]. On the theoretical side, the prevailing dipole model treats particles as point dipoles and does not consider the volume effect nor the distortion of the electric field caused by the particles [3]. The more comprehensive Maxwell stress tensor (MST) considers the effects mentioned above, but cannot predict the surface ion adsorption and the forming of an electric double layer (EDL), although an effective surface conductance can be assigned to account for the EDL effect [30,31]. More recently, Zhao et al. propose a new volumetric-integration (VI) method, which addresses those limitations and applies to particles of various sizes and shapes [31,32]. One major challenge of comparing different experimental methods and theoretical models is that the results were obtained by different research groups. Hence, the experimental/simulation conditions were often not the same. However, the particle surface property and the fluid drag force are known to greatly affect the DEP crossover frequency, and hence must be carefully considered when interpreting results obtained using different methods or different microfluidic systems. For instance, although polystyrene (PS) particles have a low material electrical conductivity, they have shown a high overall conductivity, when suspended in medium, according to dielectric measurements [25], which is evidence that the surface charge exists and is dominant. Another possible source of discrepancy in the DEP crossover frequency is the fluid motion caused by the electrohydrodynamic effect (EHD), including AC electro-osmosis (ACEO) [33] and the electrothermal effect (ETE) [26]. Unlike DEP, electrohydrodynamic forces (*F*_EHD_) cause fluid motion, which in turn results in a viscous drag on the particles [26]. Such a flow-driven particle motion couples with the pure DEP-induced particle motion, and affects the measurement accuracy of the DEP crossover frequency. The competing behaviors of ACEO and DEP have also been shown for microfluidic devices [34,35,36,37].

**Figure 1:**
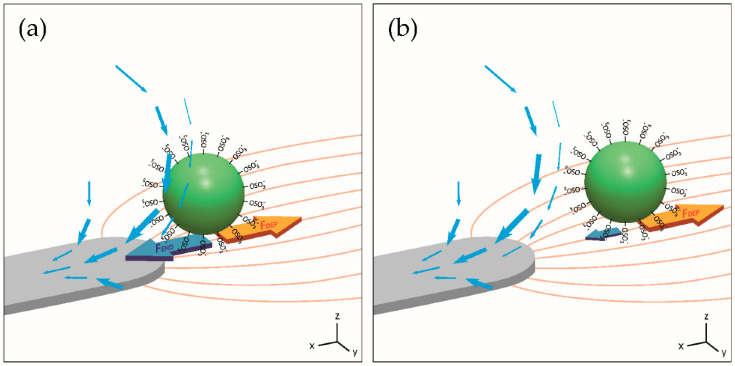
Schematic showing the concurrent forces acting on a functionalized particle in an AC electric field. The yellow curves and blue arrows indicate the electric field and the fluid flow, respectively. The size of the arrows represents the magnitude of the force and velocity. The electrohydrodynamic force is larger at the electrode edge (**a**) than that at a distance (**b**), while the dielectrophoretic (DEP) force is insensitive to the particle/electrode distance.

In this paper, we aim to provide a comprehensive direct comparison between the above-mentioned experimental methods and theoretical models under the same conditions to minimize the discrepancy due to uncertain/inconsistent material properties and electrode design. We consider a functionalized particle, located at different distances from the electrode (Figure 1). We quantify how the particle functional group and initial surface charge affect the crossover frequency. The contribution of DEP and electrohydrodynamic forces (*F*_EHD_) cannot be easily dissected experimentally. To quantify the precise effect of electrohydrodynamic forces on the DEP crossover frequency, we established finite element models using a commercial finite-element package COMSOL to elucidate the complex relationship between the DEP force and the fluid flow drag force induced by the electrohydrodynamic effect. Symbols are listed in Table 1.

## 2. Theory

### 2.1. Dielectrophoresis (DEP)

The most commonly used approaches to calculating the DEP force are the point-dipole (PD) model and the Maxwell stress tensor (MST) method. The PD model gives a closed-form expression of the DEP force, which is convenient and simple but unable to capture the distortion of the electric field due to the presence of particles. Both the PD model and the MST method account for the effect of an electric double layer (EDL) at the particle–medium interface by simply assigning a constant value of surface conductance (*K_s_*), usually an empirical value around 1 nS [23], and the overall effective total conductivity of the particle is then obtained by a simple equation ($\sigma_{\mathrm{total}}=\sigma_{\mathrm{bulk}}+2K_{s}/R$; where *R* is the particle radius and *σ*_bulk_ the bulk particle material conductivity) [23,38]. In this case, *K_s_* includes the overall effect of the Stern and diffuse layers. Although *K*_s_ can sometimes be obtained by fitting experimental data, it is not easy to predict this value before an experiment is conducted.

To address the limitations, we adopt the volumetric-integration model (VI model), developed by Zhao et al. in 2017 [31], to calculate the DEP force, considering the EDL and surface ion adsorption. This model captures the volumetric polarization of a particle by the concept that the electric field of the induced dipole in the particle equals to the difference between the electric field with and without the presence of the particle,

(1) $$E_{2}-E_{1}=-\frac{1}{2\pi \varepsilon_{m}}\frac{Q}{{\left(d/2\right)}^{2}}d=-\frac{dm}{3\varepsilon_{m}(4/3)\pi {\left(d/2\right)}^{3}}=-\frac{P}{3\varepsilon_{m}}$$

(2) $$F_{DEP}=∭(P\cdot \nabla )E_{1}dV=∭(3\varepsilon_{m}(E_{1}-E_{2})\cdot \nabla )E_{1}dV$$

where **E**_1_ is the electric field without the particle (only electrolyte), **E**_2_ is the field with the distortion induced by the presence of the particle, *ε_m_* is the permittivity of the medium, **d** is the vector distance between the negative and positive charges of the induced dipole, *d***m** is the dipole moment and **P** is the polarization density of the particle.

Furthermore, to account for the influence of the EDL structure with the actual physical mechanism of its formation, the EDL potential *φ* is determined from the Poisson–Boltzmann equation with the boundary condition at the particle–electrolyte interface in terms of the surface charge density of the particle

(3) $$-\nabla^{2}\phi =\frac{F\left[c\cdot \exp\left(-\frac{e\phi }{kT}\right)-c\cdot \exp\left(\frac{e\phi }{kT}\right)\right]}{\varepsilon_{m}}$$

(4) $$n\cdot \nabla \phi =\frac{q_{\mathrm{initial}}+q_{\mathrm{ads}}}{\varepsilon_{m}}$$

where *F* is the Faraday constant, *e* is the elementary charge, *k* is the Boltzmann constant, *T* is the temperature, *c* is the concentration of electrolyte, *q*_initial_ is the initial surface charge density and *q*_ads_ is the adsorbed surface charge density determined by the adsorption theory of Langmuir isothermal along with the Grahame EDL structure model [10], which can be written in an analytical expression of

(5) $$q_{\mathrm{ads}}=\frac{Bq_{\max}-A-1+\sqrt{{\left(Bq_{\max}-A-1\right)}^{2}+4ABq_{\max}}}{2B}$$

(6) $$A=c^{*}\cdot \gamma \cdot \exp\left(\frac{e\phi_{d}}{kT}\right)\cdot \exp\left(\frac{eq_{\mathrm{initial}}d}{\varepsilon_{i}kT}\right),\;B=\frac{eAd}{\varepsilon_{i}kT}$$

where *q*_max_ is the maximum surface charge density (the adsorption limit, simply assumed to be 10 times the initial surface charge density), c* is the dimensionless concentration, *γ* is the binding constant (according to the Gibbs free energy of ion transfer), *φ_d_* is the potential at the outer Helmholtz plane, and *ε_i_* is the permittivity between the inner and outer Helmholtz plane. Then the spatially varying conductivity at any point in the EDL can be determined through [39]:

(7) $$\sigma_{\mathrm{diffuse}}=\lambda_{+}c\cdot \exp\left(-\frac{e\phi }{kT}\right)+\lambda_{-}c\cdot \exp\left(\frac{e\phi }{kT}\right)+\frac{F\left[c\cdot \exp\left(-\frac{e\phi }{kT}\right)-c\cdot \exp\left(\frac{e\phi }{kT}\right)\right]\cdot \varepsilon_{m}\cdot (\phi -\zeta )}{\eta }$$

where λ_+_ and λ_−_ are the limiting molar conductivities ($\lambda_{+}=\lambda_{Na^{+}}=50.08\times 10^{-4}$ m^2^ S/mol and $\lambda_{-}=\lambda_{Cl^{-}}=76.31\times 10^{-4}$ m^2^ S/mol for NaCl medium) [40], *η* is the viscosity of the medium, and *ζ* is the zeta potential [39].

The Stern layer conductance, contributed by the adsorbed ions, is included by

(8) $$K_{\mathrm{stern}}=q_{\mathrm{ads}}\mu_{s}$$

where *μ_s_* is the mobility of co-ions in the Stern layer. We assume *μ_s_* = 0.5*μ_c_*, where *μ_c_* is the mobility of co-ions in the diffuse layer since the mobility in the Stern layer is known to be smaller than that in the diffuse layer [31]. In our model, we set the spatially varying particle and medium conductivities as *σ_p_* = 2*K*_stern_/*R* and *σ_m_* = *σ*_diffuse_, respectively. Therefore, the effect of EDL on the electric field distribution is inherently considered. The approach considers surface adsorption as a function of medium conductivity, and has the following merits: (i) the numerical implementation is straightforward and accurate, and (ii) the effect of the EDL can be correlated with the underlying physics including the amount of charge adsorbed and the Stern layer conductance. 

### 2.2. Electrohydrodynamics (EHD)

An AC electric field may also cause fluid motion by electrohydrodynamic effects, i.e., AC electro-osmosis (ACEO) and electrothermal effect (ETE) [41]. Different from the DEP, these electrohydrodynamic forces drive bulk fluid flow and drag particles along (Supplementary Movie S4). 

ACEO is a frequency-dependent fluid flow due to alternating electrode polarization and counter-ion accumulation [24,25,42]. It has been applied to manipulate particles in lab-on-chip devices since introduced in 1998 [24,42,43]. Studies involving ACEO flow modeling in microfluidic systems [41,44] often refer to the numerical model established by Ramos et al. in 1999 [42], which accounts for the impedance of the electrolyte by simplifying it to be discrete current flux tubes of different radii between the electrode gap and captures the position dependency and voltage drop across the electrode EDL linearly. This linearization is doubtful when it comes to the case of nonparallel and curved-edged electrode configurations. Although this model is able to present the frequency dependency of the induced flow velocity and the characteristic of having a specific optimal frequency of maximum flow velocity, it does not always give a correct order of magnitude of the velocity due to the oversimplification of the counter-ion accumulation mechanism.

Here we establish our model of ACEO flow from another aspect referring to the pioneering work by Pribyl et al. in 2008 [45]. The flow field of the electrolyte solution is governed by the Navier–Stokes momentum and continuity equations including an electric body force term (**f***_E_*),

(9) $$\rho (\frac{\partial v}{\partial t}+v\cdot \nabla v)=f_{E}-\nabla p+\eta \nabla^{2}v$$

(10) ∇⋅v=0

where *ρ*, **v**, *p* and *η* are the fluid density, flow velocity, pressure, and viscosity. The electric body force **f***_E_* equals to *ρ_v_***E**, where *ρ_v_* and **E** are the volume concentration of the mobile electric charge and the electric field, respectively, defined by $\rho_{v}\equiv F\sum_{i}z_{i}c_{i}$ and **E** ≡ −▽φ, where *z_i_*, *c_i_*, and φ denote the charge number of the *i*^th^ ionic component in the electrolyte (*i* = 2 for NaCl), the volume concentration (mol/m^3^) of the *i*^th^ ion, and the electric potential, respectively. Also,$\nabla \cdot D=\rho_{v}$, where **D** denotes the electric displacement field.

The ionic concentrations *c_i_* are determined by the molar balances without chemical reactions.

(11) $$\frac{\partial c_{i}}{\partial t}+v\cdot \nabla c_{i}=-\nabla \cdot J_{i}$$

where **J***_i_* is the sum of the local diffusive and the local electromigration flux intensities of the *i*^th^ ionic component given by the Nernst–Planck equation

(12) $$J_{i}=-D_{i}\nabla c_{i}+z_{i}D_{i}Fc_{i}E/(RT)$$

The electric potential can be determined by the Poisson equation

(13) $$\nabla^{2}\Phi =-\frac{\rho_{v}}{\varepsilon }$$

Electrothermal effect (ETE) refers to the fluid flow induced by Joule heating in the fluid surrounding the electrodes when an electric field **E** is applied [24,41]. The power generation per unit volume is given by *W* = *σ_m_ E^2^*, where *σ_m_* and *E* are the medium electrical conductivity and the magnitude of the electric field. The steady-state temperature rise can be obtained by solving the following energy balance equation

(14) $$k_{m}\nabla^{2}T+\sigma_{m}E^{2}=0$$

where *T* is the temperature and *k_m_* is the medium thermal conductivity. Note that the transient effect ($\rho_{m}c_{p}\partial T/\partial t$, where *ρ_m_* and *c_p_* are the mass density and the specific heat, respectively) and the fluid flow effect ($\rho_{m}c_{p}v\cdot \nabla T$) are in general negligible in microelectrode fluidic devices [24].

Joule heating is highly non-uniform, resulting in conductivity and permittivity gradients in the fluid. The conductivity gradient (∇σ=(∂σ/∂T)∇T) generates free volume charge and the Coulomb force, whereas the permittivity gradient (∇ε=(∂ε/∂T)∇T) generates the dielectric force. These forces cause fluid motion. The total force per unit volume can be written in the form of the time-averaged Korteweg–Helmholtz force density equation for incompressible flow [24,41,44],

(15) 〈fE〉=12Re[((σ∇ε−ε∇σ)⋅Eσ+jωε)E*−12|E|2∇ε]

where *ω* is the angular frequency of the applied electric field. 

## 3. Materials and Methods 

### 3.1. Experimental Setup: Optical Tweezers and a Microfluidic Chip

**Figure 2:**
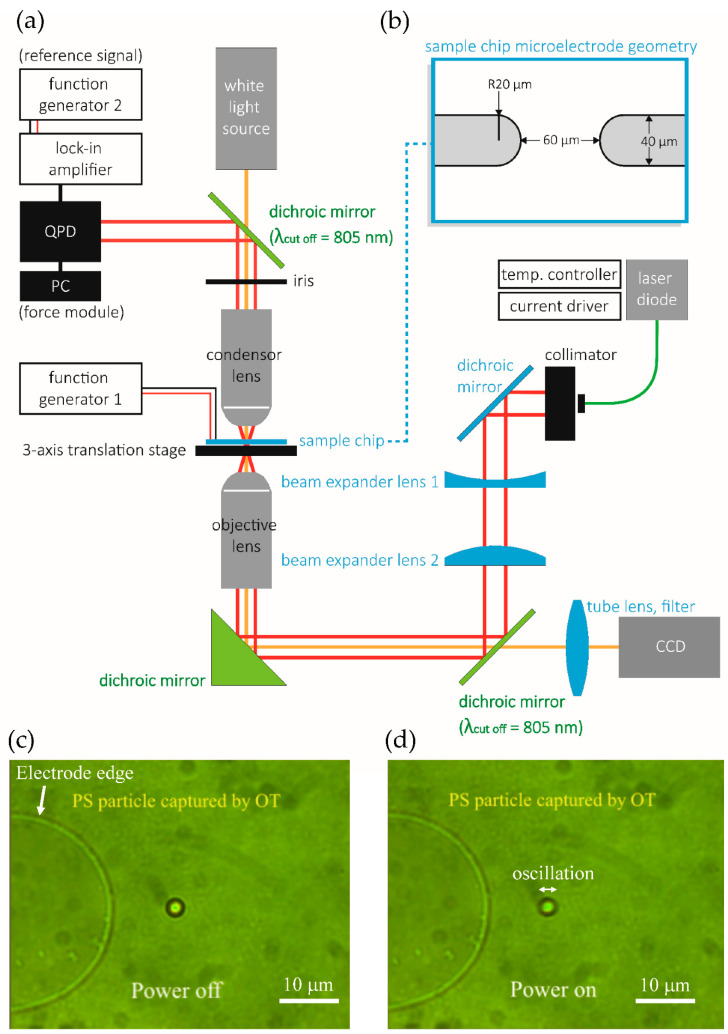
(**a**) Experimental apparatus, (**b**) microfluidic chip, and representative images of a particle trapped by optical tweezers (**c**) with AC power off (no DEP force)—the particle is stationary—and (**d**) with AC power on—the particle oscillates at the amplitude modulation (AM) frequency of 1 Hz with an amplitude of approximately ±1 μm. (See the Supplementary Movie S1).

Figure 2 shows the schematic diagram of our experimental apparatus, which includes a simple microfluidic chip, a lock-in amplifier, a quadrant photodiode (QPD), and an optical tweezer (OT) system constructed using commercially available optical and mechanical components from Thorlabs. We use a distributed feedback laser with a wavelength of 975 nm and a maximum power of 330 mW, which delivers a resolution of ~0.05 pN, a spot size of ≥0.6 μm, and a maximum trapping force of ~20 pN. The displacement of a trapped particle is measured by the QPD, which was first calibrated using a three-axis piezoelectric sample positioning stage (NanoMax^TM^ Stage, Thorlabs). The tweezer stiffness, *k*, is calibrated to be ~1.3 × 10^−5^ N/m at 120 mW. We fabricated a simple microfluidic chip with pairs of microelectrode arrays by patterning a slide glass coated with 220-nm indium tin oxide (ITO). Each electrode is approximately 40 μm wide, and the distance between a pair is 60 μm. Deionized water containing polystyrene (PS) particles is dispensed on the electrode arrays using a micropipette, and a cover glass is then used to cover the colloid solution and electrodes. The gap between the cover glass and the slide is 60 μm. Thus the liquid is contained in a chamber (dimension: 20 mm × 20 mm × 60 μm). Four different sizes of functionalized and nonfunctionalized particles are studied (1, 2, 3, and 4 μm in diameter, Molecular Probes^®^). Each batch comes with a certificate of analysis showing a detailed lot of data, including the important initial surface charge density (Table 2). The medium conductivity, ranging from 1.0 × 10^−4^ S/m to 1.0 × 10^−3^ S/m), is adjusted by NaCl concentration. Such a parameter range is chosen since it is where most discrepancies occur [23,29,30,31]. A representative process of how the optical tweezers work is shown in Supplementary Movie S2.

This apparatus is used to measure the crossover frequency by using three different methods: (1) the observation method, (2) the direct OT force method, and (3) the phase shift method. In the observation method, the crossover frequency is determined by visually observing the change of direction of the particles without the use of the optical tweezers (Supplementary Movie S3). In the direct OT force method, the DEP force applied on an OT-trapped particle is measured by the spring equation, *F*_OT_ = *F*_DEP_ = *kx*, where *x* is the lateral displacement of the trapped particle from the beam focus [27,46]. In the phase-shift method, we follow the work by Wei et al. [29] but use only one OT laser, which retains the same accuracy with a much simpler calibration process. We determine the point of crossover at the instant that the oscillation point of the particle undergoes a sudden displacement (Figure 2c,d), which produces a phase shift signal. This signal indicates a directional reversal of the resultant—both DEP and electrohydrodynamic forces—exerted on a particle captured with the OTs. Note that if the electrohydrodynamic force exists and is not negligible, the measured (perceived) crossover frequency may considerably deviate from the actual DEP crossover frequency. In addition, we conducted our frequency sweep from high frequencies (~10 MHz) toward low frequencies (~10 KHz) in order to minimize the influence of strong ACEO flow on crossover frequency in the low-frequency regime.

### 3.2. Numerical Simulations by Finite Element Analysis (FEA)

**Figure 3:**
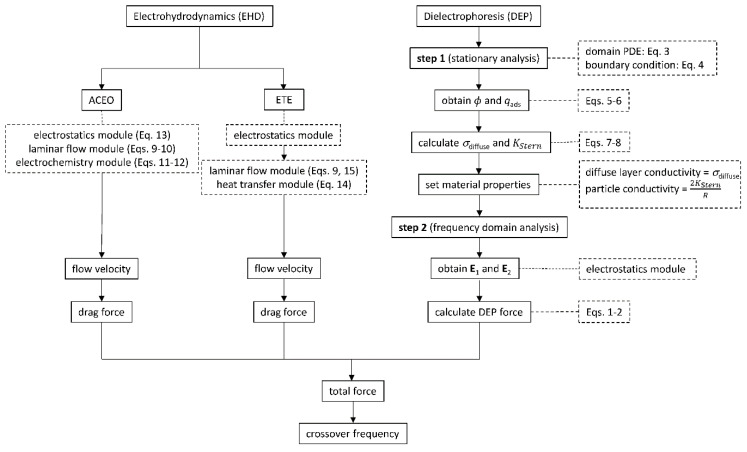
Flowchart of finite element analysis (FEA) simulations for determining the DEP force and the perceived DEP crossover frequency if the electrohydrodynamic flow exists.

Numerical implementation of the mathematical models of DEP, ACEO, and ETE is performed using the commercial finite-element package COMSOL. The flowchart of the entire simulation with the corresponding equations is shown in Figure 3. The DEP, ACEO, and ETE simulations are carried out separately in three uncoupled models. For the DEP, we use a two-dimensional (2D) axisymmetric model similar to Ref. [31]. The domain is 5*R* × 3*R*. The top electrode is biased by an AC signal of 5 V, i.e., *V_pp_* = 10 V; the bottom electrode is grounded. As described in the Theory section, the EDL is modeled by setting *σ_p_* = 2*K*_stern_/*R* and *σ_m_* = *σ*_diffuse_. Note that, since *σ*_diffuse_ decreases dramatically with the distance from the particle, its effect is mostly within a thin layer outside the particle. Also, since the Stern layer thickness is always negligible compared with the particle size, the layer is not physically modeled. However, its effect on the electric field and particle conductivity is included. 

**Figure 4:**
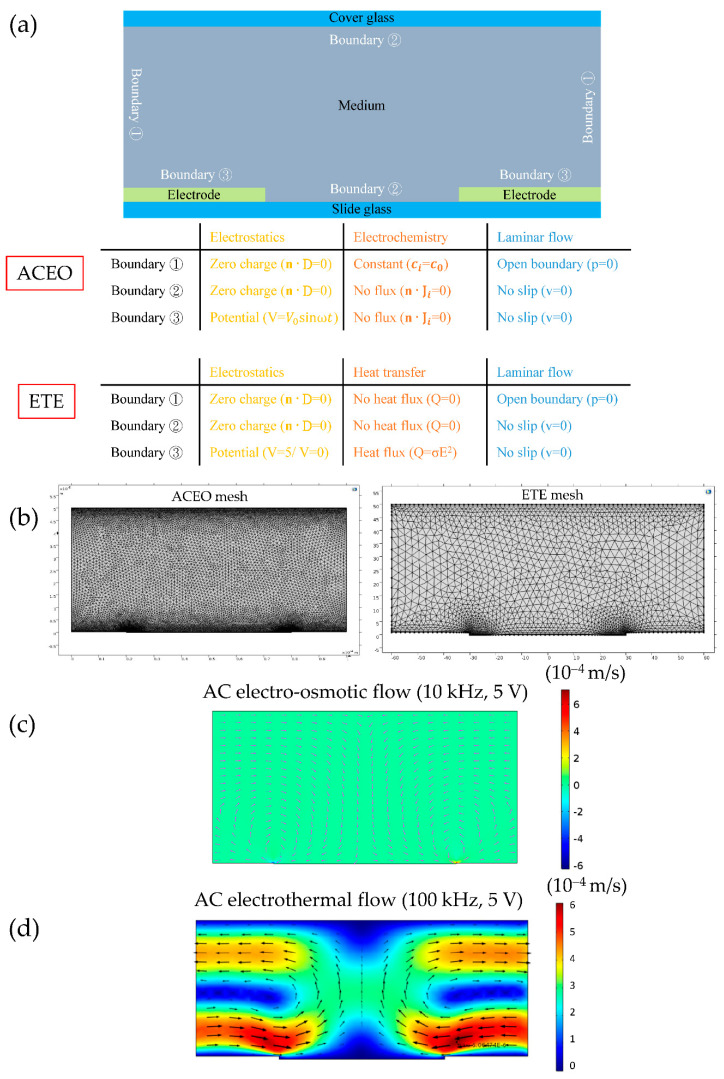
FEA simulations of AC electro-osmosis (ACEO) and electrothermal effect (ETE) velocity fields using the commercial package COMSOL. (**a**) Computational domain and boundary conditions. The constant c0 is calculated by *σ_m_* = *c_0_* (λ_+_ + λ_−_), where λ_+_ and λ− are the limiting molar conductivities of Na^+^ and Cl^−^. (**b**) Representative meshes for ACEO (left panel) and ETE (right panel), respectively. (**c**) A representative instantaneous velocity field of ACEO at 10 kHz and 5 V. *σ_m_* = 1 × 10^−3^ S/m. (**d**) A representative average (stationary) velocity field of ETE at 100 kHz and 5 V. *ο_m_* = 10 × 10^−4^ S/m. The heat transfer between the cover glass/slide glass and the fluid is considered.

For the ACEO simulation, we solve the 2D nonstationary Poisson–Nernst–Planck–Navier–Stokes equations by coupling three modules: transport of diluted species (tds), electrostatics (es), and laminar flow (spf). For the ETE, we carry out 2D stationary simulation by coupling three modules: electrostatics (es), heat transfer in fluids (ht), and laminar flow (spf). The electric field and heat generation are first solved by the electrostatics module. Then the temperature and flow velocity are solved simultaneously by coupling the heat transfer and laminar flow modules, using the time-averaged Korteweg–Helmholtz force density equation—Equation (15)—as the body force. The computational domain and boundary conditions, a representative mesh, a representative AC electro-osmotic instantaneous velocity field (10 kHz), and a representative AC electrothermal average (stationary) velocity field (100 kHz) are shown in Figure 4a–d. In the electro-osmosis simulations, the EDL effect is considered. This means that the required characteristic dimension of the finite elements at the electrode surfaces typically differs from the element size in the rest of the domain of the microfluidic chip by four orders of magnitude. It can only be partially resolved by constructing a non-uniform mesh, which is finer near the electrode surface. It is almost impossible to explicitly mesh the nanometer-sized Stern layer and inaccuracy to some degree is inevitable. In the present case, we used the “extra-fine” mesh setting in COMSOL, which created two thin layers of rectangular elements along the electrode surface (Figure 4b, left panel). The thickness of those rectangular elements is approximately 20 nm. We estimate that the Debye length, where the cation and anion concentrations are equal, is ~100 nm for the case in Figure 4c. The normal extent of the EDL is approximately equal to the Debye length.

In this paper, we separately consider three effects, and for each of them we perform a dedicated run instead of performing multiphysics simulations including all the relevant effects in a single run. We assume that the superposition principle holds. However, it is in principle possible to run a complete simulation and decompose the action in several contributions. More future studies are required to quantify how much error this approximation may introduce.

## 4. Results and Discussion

### 4.1. Direct Comparison of Three Experimental Methods

**Figure 5:**
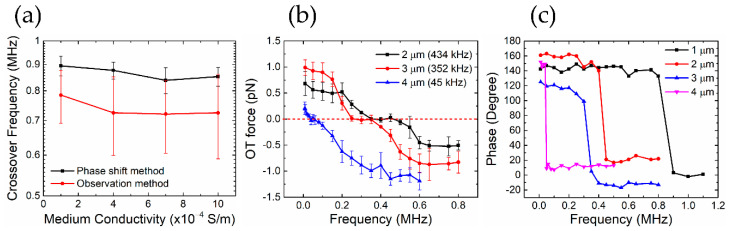
Comparison of crossover frequency measured by three methods. (**a**) Observation method (particle diameter: 1 μm); (**b**) direct optical tweezers (OT) force method (particle diameters: 2 μm, 3 μm, and 4 μm); (**c**) lock-in amplifier aided phase shift method (particle diameters: 1 μm, 2 μm, 3 μm, and 4 μm). All particles are sulfate-functionalized polystyrene (PS). The error bars represent one standard deviation of 15 repeated measurements.

Figure 5 shows a direct comparison of the crossover frequency obtained by three experimental methods. The crossover frequency obtained by the observation method exhibit larger standard deviations and is in general smaller in magnitude (~15%) than those obtained by the more accurate phase shift method (Figure 5a). Another major problem of the observation method is that it is difficult, if not impossible, to isolate a single particle, hence the measured frequency is questionable due to the effect of particle–particle interaction [32]. As to the direct OT force method, there is a range of several hundred kHz where the measured forces are approximately zero—a clear-cut crossover frequency is too challenging to obtain since the force in this range is extremely small (~0.1 pN). However, the phase shift data converted from the lock-in amplifier does provide a considerably clear definition of the crossover frequency (Figure 5c). We conclude that the phase shift method is the most accurate and repeatable. All the experimental data presented here were obtained using this method unless stated otherwise.

**Figure 6:**
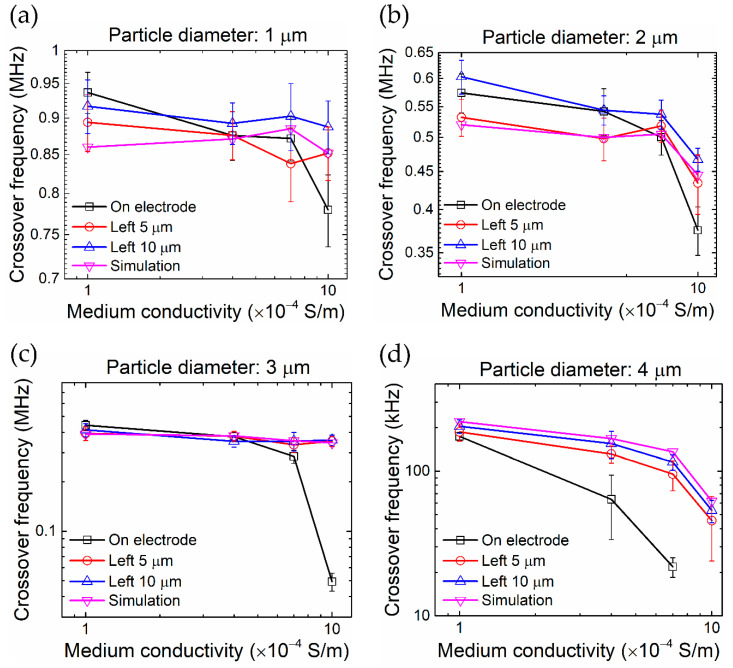
Effect of particle–electrode distance on the measured (perceived) crossover frequency. (**a**) Particle diameter of 1 μm, (**b**) 2 μm, (**c**) 3 μm, and (**d**) 4 μm. “On electrode”, “Left 5 μm”, and “Left 10 μm” indicate that the particle–electrode distance is 0, 5 μm, and 10 μm, respectively. “Simulation” indicates the simulated DEP crossover frequency, which is not sensitive to the particle location, without the electrohydrodynamic effect. Note that the measured crossover frequencies of the “On electrode” condition exhibit significant deviations from the other cases due to the influence of the electrohydrodynamic flow, and hence cannot be interpreted as the DEP crossover frequencies. The error bars represent one standard deviation of 15 repeated measurements.

Moreover, we observed that the particle–electrode distance plays a key role in the determination of DEP crossover frequency. Figure 6 shows a comparison of measured crossover frequency as a function of particle size, medium conductivity, and particle–electrode distance. When placed at the edge of the electrode, the particle exhibits a much lower crossover frequency, compared to that measured at a distance from the electrode, especially in the medium of conductivity higher than 7 × 10^−4^ S/m. Such a deviation is much more pronounced for larger particles, e.g., diameter > 3 μm. Also, the frequency measured at the edge of the electrode does not agree with the simulated value obtained by the pure DEP finite element model (VI model), suggesting that it is deficient in predicting the particle motion at such a critical position by assuming that DEP force is dominant and other concurrent effect is negligible.

### 4.2. Flow Velocity Due to ACEO and ETE

**Figure 7:**
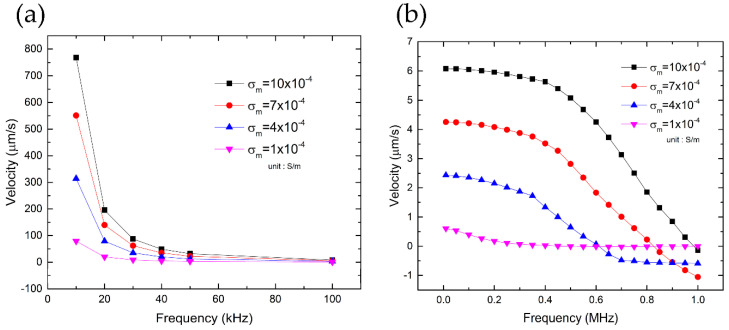
(Simulation) velocity of fluid flow near the electrode edge as a function of applied frequency and medium conductivity. (**a**) ACEO effects are more dominant with higher medium conductivities and lower applied frequencies. (**b**) ETE flow velocity has a lower dependence on applied frequency in comparison with ACEO flow but also raises with medium conductivity.

The flow velocity field near the electrode edge is simulated and shown in Figure 7. Within a specific range of frequencies and conductivities, these flows can exert a drag force (the Stokes’ drag law, $f_{\mathrm{drag}}=6\pi \eta Rv$ [44]) of the same order of magnitude as the DEP force on the microparticle (especially for the larger ones since **f**_drag_ ∝ *R*) held at the electrode edge by the optical tweezers. This is consistent with the experimental observation as shown in Figure 6, and is important to be taken into account to obtain an accurate analysis of the DEP phenomenon and the crossover behavior of particles. Note that the Stokes’ drag law is valid only if the velocity field can be assumed uniform on the length scale of the particle diameter. From Figure 4c,d, we observed that the ACEO velocity field is much more non-uniform than that of the ETE flow. Thus, we expect that the Stokes’ law is more applicable to the ETE and 1 μm particles than the ACEO and 4 μm particles. Here, we used the velocity at the particle center to calculate its drag force, which we believe is a good approximation for capturing the first-order effect. However, more future studies are required to quantify how much error this approximation may introduce.

### 4.3. Contribution of Various Forces on the Measurements of Crossover Frequency

**Figure 8:**
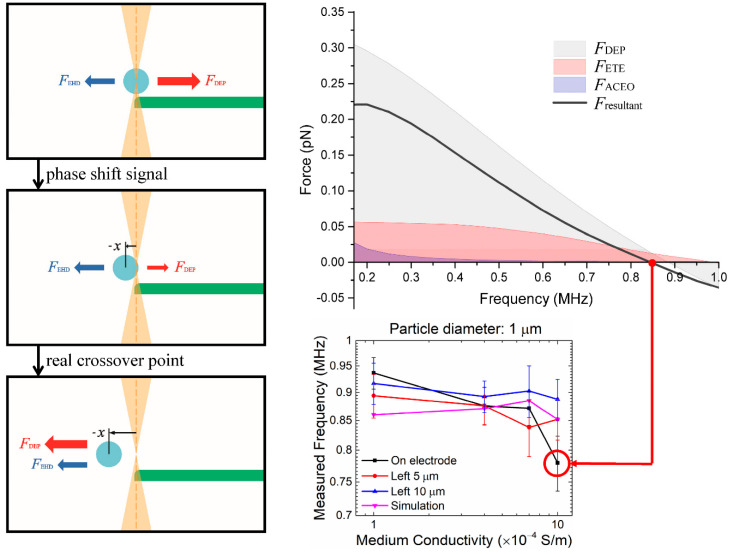
Stack plot of the individual DEP, ACEO, and ETE forces and their resultant exerted on a 1 μm sulfate-functionalized particle placed at the electrode edge in a NaCl electrolyte of conductivity *ο_m_* = 1 × 10^−3^ S/m. The measured crossover frequency (at *F*_resultant_ = 0) is much lower than the actual DEP crossover frequency (at *F*_DEP_ = 0) due to the influence of electrothermal force *F*_ETE_. The error bars represent one standard deviation of 15 repeated measurements.

**Figure 9:**
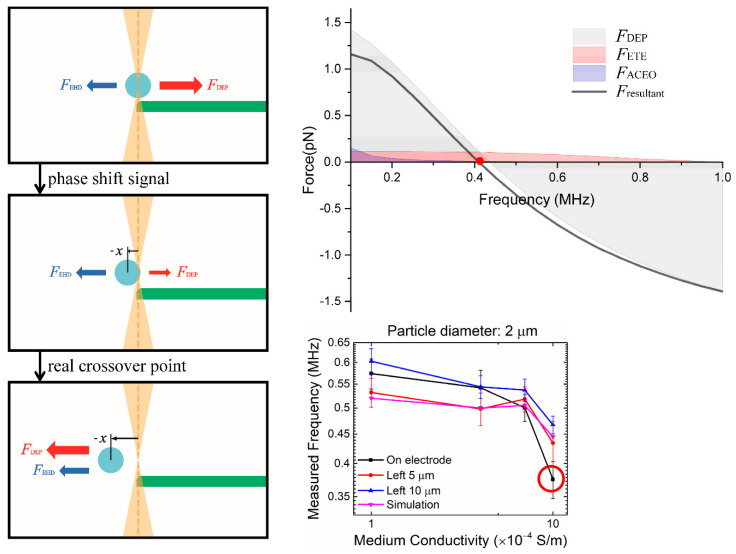
Stack plot of the individual DEP, ACEO, ETE forces and their resultant exerted on a 2 μm sulfate-functionalized particle placed at the electrode edge in a NaCl electrolyte of conductivity *σ_m_* = 1 × 10^−3^ S/m. The measured crossover frequency (at *F*_resultant_ = 0) is much lower than the actual DEP crossover frequency (at *F*_DEP_ = 0) due to the influence of electrothermal force *F*_ETE_. The error bars represent one standard deviation of 15 repeated measurements.

**Figure 10:**
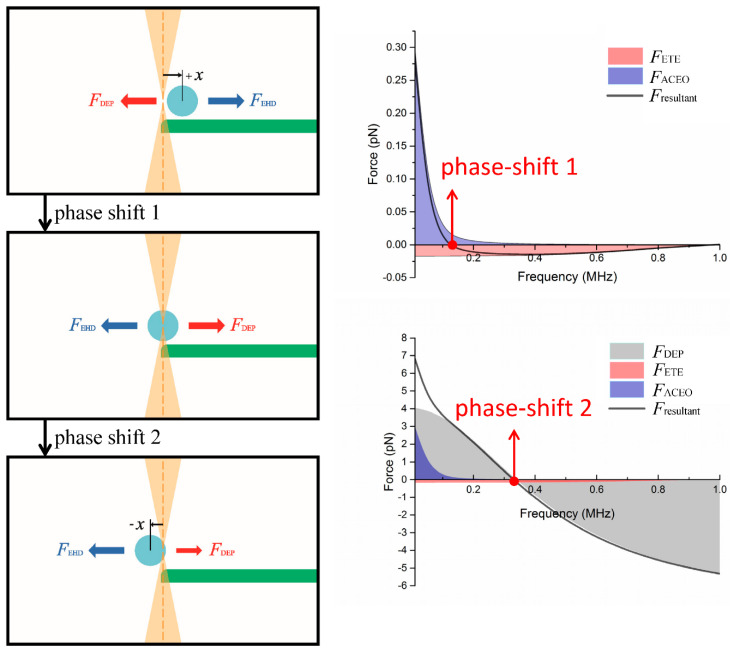
Stack plot of the individual DEP, ACEO, ETE forces and their resultant exerted on a 3 μm sulfate-functionalized particle placed at the electrode edge in NaCl electrolyte of conductivity *σ_m_* = 1 × 10^−3^ S/m. Two phase-shift signals were measured at ~120 kHz and ~340 kHz; the smaller one is due to the balance of electro-osmosis force (*F*_ACEO_) and electrothermal force (*F*_ETE_), and hence should not be misinterpreted as a DEP crossover frequency.

**Figure 11:**
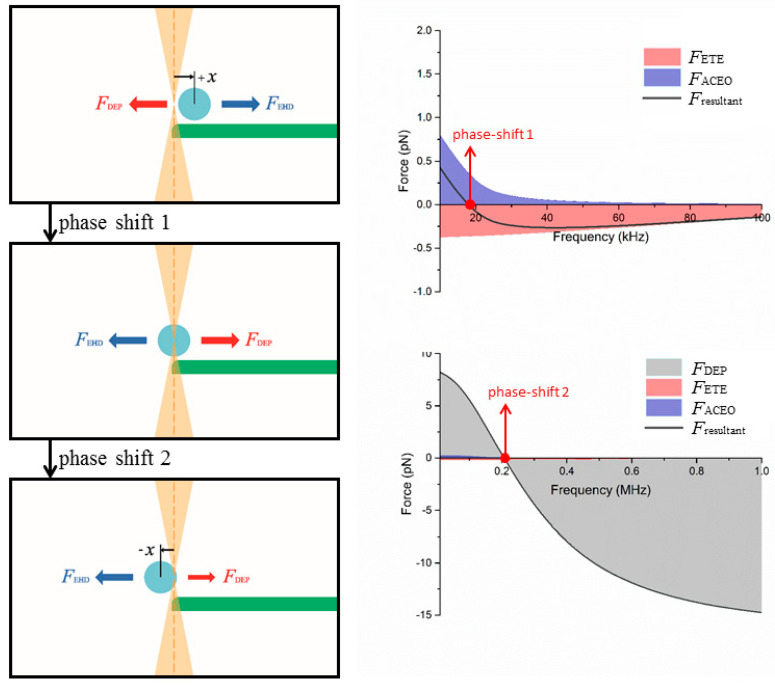
Stack plot of the individual DEP, ACEO, ETE forces and their resultant exerted on a 4 μm sulfate-functionalized particle placed at the electrode edges in a NaCl electrolyte of conductivity *σ_m_* = 7 × 10^−4^ S/m. Two phase-shift signals were measured at ~20 kHz and ~200 kHz; the smaller one is due to the balance of electro-osmosis force (*F*_ACEO_) and electrothermal force (*F*_ETE_), and hence should not be misinterpreted as a DEP crossover frequency.

Using the experimental and simulation results, we performed a comprehensive analysis of the crossover behavior and identified the contribution of various forces on the measurements of crossover frequency. Particles at the electrode edge and in relatively high medium conductivity, e.g., *σ_m_* = 1 × 10^−3^ S/m, tend to exhibit a measured crossover frequency much lower than the theoretical value (Figure 6). We performed simulations to obtain the DEP, ACEO, ETE forces, and their resultant exerted on a particle held near the electrode edge in a NaCl electrolyte (Figure 8, Figure 9, Figure 10 and Figure 11).

Small and large particles exhibit different behaviors and are thus discussed separately. For smaller particles, i.e., *R* = 0.5 μm and 1.0 μm, when we sweep the AC frequency from 100 kHz to 1 MHz, the ACEO drag force is apparently smaller than the other two forces and also decreases rapidly as the frequency approaches the crossover point (Figure 8 and Figure 9). In this case, the crossover behavior is mainly determined by the DEP force and the ETE drag force. Figure 8 is the stack plot of forces exerted on a 1 μm particle at the electrode edge in NaCl electrolyte (*σ_m_* = 1 × 10^−3^ S/m). At lower frequencies under 500 kHz, the positive DEP (pDEP) force is much higher than the electrohydrodynamic drag forces, and thus the pDEP force (toward the edge of the electrode) dominates the point of particle oscillation [29], which is at the center of the OTs (that coincides with the edge of the electrode) at this instance. As we increase the AC frequency up to about 800 kHz, the pDEP force reduces to the order of 10^−14^ N, which is the same as the order of the ETE force (−*x* direction) in this range of AC frequency. Then, for an AC field of 845 kHz, the resultant force and the corresponding offset point of oscillation goes through a sudden shift (from 0 to −*x*) and causes a QPD lock-in output of a sharp phase shift. This phase shift is commonly misunderstood as the DEP crossover point but is actually a value with error due to the influence of ETE flow. Thus, we conclude that the experimental data of phase-shifting frequency obtained from particles held at the electrode edge should not be defined as the crossover frequency, and the actual DEP crossover frequency should be the higher ones measured 10 μm away (≈17% of the electrode–electrode gap) from the electrode edge (887 kHz), where the influence of the ETE flow is minimum. As for the behavior of 2 *μ*m particles, the mechanism is the same as the 1 μm ones, and its corresponding stack plot is shown in Figure 9. The directional reversion of the resultant force is around 400 kHz and the actual DEP crossover frequency should be ~15 kHz higher.

For larger particles (3 μm and 4 μm in diameter), their crossover phenomenon is different from that of the smaller ones, mainly because larger particles possess lower crossover frequencies where the ETE and ACEO flow velocities are higher and also these particles receive larger drag forces due to their larger size. Figure 10 is the stack plot of the forces exerted on a 3 μm particle at the electrode edge in NaCl electrolyte (*σ_m_* = 1 × 10^−3^ S/m). When we sweep the AC frequency from 10 kHz to 200 kHz, we receive a phase-shift signal at around 120 kHz, denoted phase-shift 1. This phase-shift is not a crossover point of the DEP force but a directional reversion of the resultant electrohydrodynamic force. Before this reversion, the electrohydrodynamic force is in the +*x* direction, so is the corresponding offset point of oscillation, which causes the direction of the pDEP force (toward the edge of the electrode) to be in the −*x* direction at this instance. As the ACEO drag decreases with the increase of frequency, the resultant electrohydrodynamic force changes from the +*x* into −*x* direction, and also the magnitude drops to near zero. As a result, the corresponding point of oscillation shifts from a +*x* offset to the center of the OTs and produces the signal of the first phase-shift. As to the second phase-shift at ~340 kHz, the mechanism is similar to that of the small particles described earlier, which is a value slightly lower than the actual DEP crossover frequency. Similar conditions for the 4 μm particles with the first phase shift ~20 kHz and the second phase-shift ~200 kHz are shown in Figure 11. In this case, a medium conductivity of *σ_m_* = 7 × 10^−4^ S/m is used (instead of *σ_m_* = 1 × 10^−3^ S/m), since there is no DEP crossover in such a condition (pDEP at any frequency).

### 4.4. Specific Ion Adsorptions

Our experimental and simulation results, e.g., “Left 10 μm” and “Simulation” in Figure 6, show that the DEP crossover frequency of a particle with a diameter from 1 μm to 4 μm decreases as the medium conductivity increases. Such medium conductivity dependency becomes more pronounced and complex for smaller particles, e.g., diameter 0.093 μm to 0.557 μm, as shown in Green et al. [25]. This is because the specific co-ion adsorption phenomenon, and hence the effect of EDL, is much more evident on smaller particles [31].

**Figure 12:**
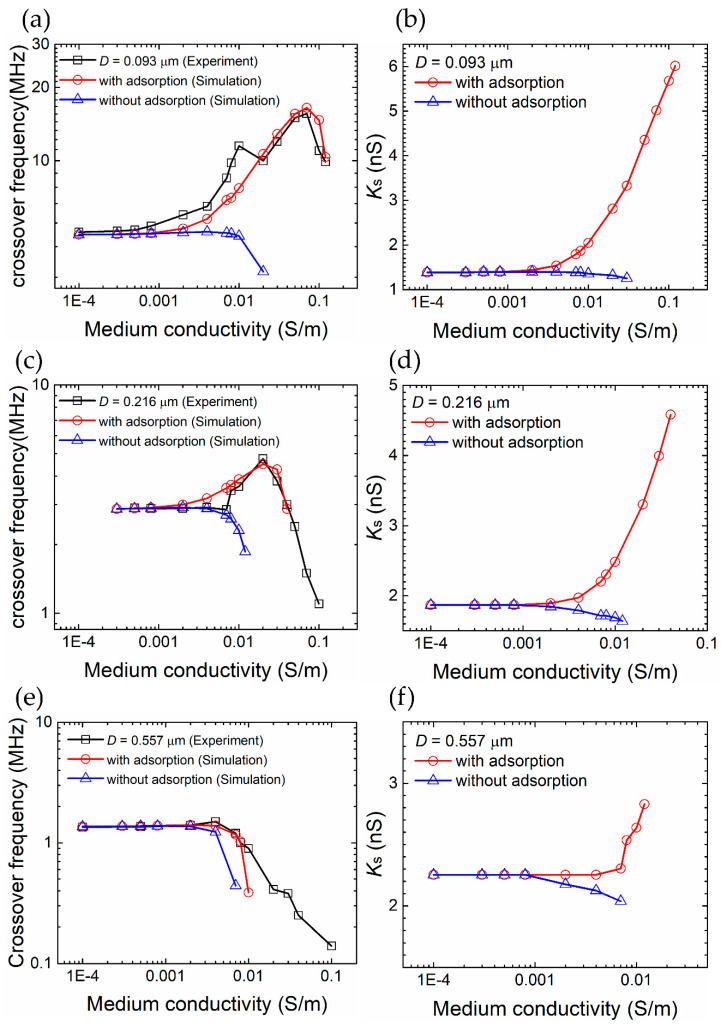
Specific ion adsorption and the electric double layer (EDL) have significant effects on the crossover frequency and surface conductance in particular for small particles (diameter = 0.093 μm) in high medium conductivity conditions. (**a**,**c**,**e**) Comparison of our volumetric-integration simulations, with and without ion-adsorption, and measurements from [25]. (**b**,**d**,**f**) Comparison of the fitted surface conductance *K_s_* used in our MST simulations between cases with and without ion-adsorption.

To further elucidate this phenomenon, we performed simulations for carboxyl-functionalized particles with and without considering the co-ion adsorption. Figure 12a,c,e are the comparison of the crossover frequency between the experimental data from [25] and our simulations using the VI model. An investigation of the results highlights three key observations. First, the simulations agree well with the measurements when the co-ion adsorption is included. Second, the crossover frequency is significantly underestimated if the co-ion adsorption effect is turned off in the simulations. Third, the effect of co-ion adsorption reduces as the particle size increases or the medium conductivity decreases.

Figure 12b,d,f show the corresponding surface conductance *K_s_* used in the MST model to match the crossover frequencies determined by the VI model with and without the adsorption. We observe that the phenomenon of rising surface conductance with the medium conductivity becomes less obvious and less important as the particle size increases. In other words, because the particle diameter is larger than 0.557 μm and the medium conductivity is lower than 1 × 10^−3^S/m in our experimental setup, we can conclude that it is appropriate to set a constant value of *K_s_* in our MST model to obtain a reasonably accurate crossover frequency. As to the specific value of this constant *K_s_* will be discussed in Section 4.6.

### 4.5. Functional Groups

**Figure 13:**
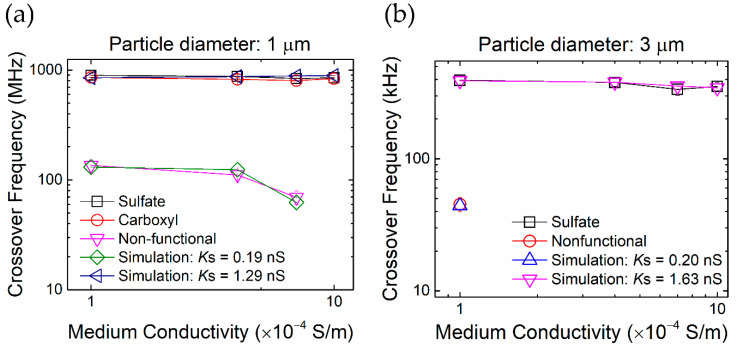
The presence of surface functional groups dramatically affects the crossover frequencies of particles. The results for sulfate and carboxyl are similar. (**a**) Crossover frequencies of 1 μm particles with different surface functional groups as a function of medium conductivity. (**b**) The case for 3 μm particles.

To understand the influence of surface functional groups on surface conductance and the crossover frequency of particles, we conducted measurements of different functionalized and nonfunctionalized particles. Figure 12a shows the measured crossover frequencies of sulfate-, carboxyl- and non-functionalized particles with a diameter of 1 μm. (There are no crossover for a medium conductivity higher than 1 × 10^−3^ S/m, where the only negative DEP (nDEP) exists under all AC frequencies.) The DEP crossover frequency of sulfate and carboxyl functionalized particles are similar and can be matched with an MST model of *K_s_* = 1.29 nS. The values of the nonfunctionalized ones are much lower and the corresponding *K_s_* is only 0.19 nS. We conclude that the presence of sulfate and carboxyl functional groups gives rise to a negative surface charge density and surface conductance as predicted by the surface charge density calculation considering the co-ion adsorption mechanism. In other words, the surface charge density and surface conductance are low if the particle is not functionalized and holds weak co-ion adsorption. The same conclusion can be obtained from Figure 13b, which is the measured crossover frequencies of sulfate and non-functionalized particles of a diameter of 3 μm. (For particles larger than 3 μm, there are no crossover points of DEP force in a medium of conductivity higher than 4 × 10^−4^ S/m, where the only nDEP exists under all AC frequencies.)

### 4.6. Surface Conductance and Initial Surface Charge Density

In this section, we further discuss a possible reason that may explain why disagreement of experimental data from different research groups may still exist, although various modifications of the surface conductance equations have been introduced [22,25,38]. Table 3 shows the corresponding values of *K_s_* to be set in an MST model to match our measured crossover frequency in each condition. In general, there are not many variations for a given particle size across this medium conductivity range, except for the 3 μm particles, whose *K_s_* is considerably larger than the others. We examined the initial surface charge density, provided by the vendors, of those as-fabricated particles, and found that the value for the 3 μm is significantly larger than that of other particles (Table 2). The initial surface charge density may vary from batch to batch, even for particles of the same size and functional group. Table 2 also lists the initial surface charge density (a fitting parameter for the VI model) and surface conductance (for the MST model) required for the measured crossover frequencies. The values of the 3 μm particle are relatively larger, and both models are consistent with the experiments. The as-fabricated initial surface charge density is one of the most important properties when performing DEP calculations and interpreting experimental data.

## 5. Conclusions

We present a comprehensive study to elucidate the effect of concurrent electrohydrodynamic forces on the measurement of DEP crossover frequency using the recently developed experimental phase-shift method and numerical volumetric-integration model. Compared to the conventional observation method and the more advanced direct OT force method, the phase shift method provides much higher sensitivity and accuracy. Unlike the classic dipole model and the more established MST model, the VI model considers the influence of the EDL structure by simulating the surface adsorption of co-ions without the need to assume the (unknown) surface conductance *K_s_*. We showed that the as-fabricated initial surface charge density, usually available from vendors, has a significant effect on the crossover frequency, and functionalized particles exhibit much higher surface charge than non-functionalized ones, resulting in a higher crossover frequency. Our simulations showed that electrohydrodynamic forces, such as ACEO and ETE, can apply considerable force on an OT-trapped particle, which introduces error in the measurement of crossover frequency. This effect is more pronounced if the measurement is done on particles located near the electrode edge, and for larger particles and higher medium conductivities. With this understanding in mind, we were able to measure the crossover frequency in a way that the electrohydrodynamic forces are indeed negligible. This work presents a combined experimental and numerical studies on particle DEP crossover behavior. It provides a solid foundation for understanding the complicated DEP phenomena and provides new insights for advancing new applications using DEP in lab-on-chip systems. In the future, we plan to use the methodology developed here to study the crossover frequencies of particle chains and nonspherical particles. This is important since DEP experiments are generally not conducted using single particles, and particles are known to form pearl chains at the vicinity of electrodes. Also, many natural particles and cells are nonspherical [47,48].

## Figures and Tables

**Table 1:** Nomenclature.

Notation	
*c*	bulk concentration of electrolyte (mol/m^3^)
c*	dimensionless concentration
$c_{i}$	molar concentration of the *i*^th^ ionic component (mol/m^3^)
$c_{p}$	specific heat capacity (J/kg·K)
*d*	distance of electric dipole (m)
**d**	vector distance of electric dipole (m)
*d* **m**	dipole moment (C·m)
*D_i_*	ion diffusion coefficient of the *i*^th^ ionic component (m^2^/s)
*e*	elementary charge
**E**	electric field (V/m)
F	Faraday constant
*F*_ACEO_	AC electro-osmosis force (N)
*F*_DEP_	dielectrophoresis force (N)
*F*_EHD_	electrohydrodynamic force (N)
*F*_ETE_	electrothermal force (N)
*F*_OT_	optical tweezer force (N)
$f_{drag}$	fluid drag force on particles (N)
$f_{E}$	electric body force (N/m^3^)
$J_{i}$	local ionic concentration flux of the *i*^th^ ionic component (mol/m^3^·s)
k	thermal conductivity (J/m·s·K)
*K_s_*	surface conductance (S), a fitting parameter for EDL
*K*_stern_	Stern layer conductance (S)
**P**	polarization density of particle (C/m^2^)
p	fluid flow field pressure (Pa)
*q*_ads_	adsorbed surface charge density (C/m^2^)
*q*_initial_	initial surface charge density (C/m^2^)
*q*_max_	maximum surface charge density (C/m^2^)
*R*	particle radius
*T*	temperature
**v**	fluid flow velocity (m/s)
$z_{i}$	charge number of the *i*^th^ ionic component in the electrolyte
*γ*	binding constant
ω	angular frequency of the applied AC electric field (π·Hz)
$\varepsilon_{i}$	permittivity between inner and outer Helmholtz plane
$\varepsilon_{m}$	medium permittivity (F/m)
*σ_m_*	medium conductivity (S/m)
*σ_p_*	effective particle conductivity (S/m)
*σ*_bulk_	bulk particle material conductivity (S/m)
*σ*_diffuse_	diffuse layer conductivity (S/m)
*σ*_total_	effective particle total conductivity (S/m); *σ*_total_ = *σ*_bulk_ + 2*K*_stern_/*R*
Φ	applied AC electric field electric potential (V)
ϕ	electric potential (V)
$\phi_{d}$	outer Helmholtz plane electric potential (V)
ζ	zeta potential (V)
ρ	fluid density (kg/m^3^)
$\rho_{v}$	charge volume concentration (C/m^3^)
η	viscosity (Pa·s)
*μ*	ion mobility (m^2^/V·s)
*μ_c_*	co-ion mobility in the diffuse layer (m^2^/V·s)
*μ_s_*	co-ion mobility in the Stern layer (m^2^/V·s)
λ	equivalent ionic conductivities (m^2^·S/mol)

**Table 2:** Comparison of various key parameters between sulfate-functionalized and non-functionalized particles. (Medium conductivity *σ_m_* = 1 × 10^−3^ S/m).

Functional Group	Sulfate-Functionalized				Nonfunctionalized	
Particle diameter (μm)	1	2	3	4	1	3
Surface conductance, *K_s_* (MST model) (nS)	1.29	1.44	1.63	1.05	0.19	0.20
Initial surface charge density (VI model) (μC/cm^2^)	−1.74	−1.95	−2.16	−1.40	−0.0037	−0.0037
Initial surface charge density (from vendors) (μC/cm^2^)	−1.90	−2.50	−6.70	−1.30	N/A	N/A
Adsorbed surface charge density (VI model) (μC/cm^2^)	−0.00209	−0.00152	−0.00113	−0.00385	−0.0645	−0.0637
Simulated crossover frequency (VI and MST) (kHz)	882	528	410	203	131	44
Measured crossover frequency (phase shift method) (kHz)	894	532	393	186	136	45

**Table 3:** Surface conductance *K_s_*, used in the Maxwell stress tensor (MST) simulations, to obtain the same crossover frequency as measured by the phase shift method for each particle diameter under different medium conductivities. The particles are sulfate-functionalized.

Particle Diameter	Medium Conductivity			
	1 × 10^−4^ S/m	4 × 10^−4^ S/m	7 × 10^−4^ S/m	1 × 10^−3^ S/m
1 μm	1.33	1.29	1.27	1.27
2 μm	1.49	1.43	1.45	1.38
3 μm	1.75	1.65	1.55	1.58
4 μm	1.15	1.00	1.03	1.03

## Supplementary Materials

The following are available online at https://www.mdpi.com/2079-4991/10/7/1364/s1, Video S1: DEP oscillation, Video S2: Brownian motion and optical tweezers, Video S3: From pDEP to nDEP, Video S4: ACEO.
