# Supplementary figures and images for: Dielectrophoretic Crossover Frequency of Single Particles: Quantifying the Effect of Surface Functional Groups and Electrohydrodynamic Flow Drag Force
Source: Nanomaterials (Basel). 2020 Jul 13;10(7):1364. doi: 10.3390/nano10071364 (PMC7408174; doi:10.3390/nano10071364)

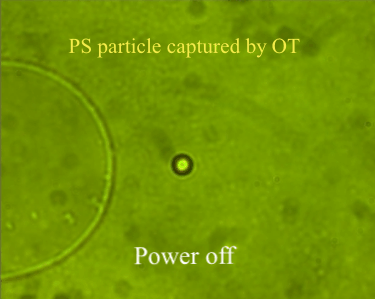

Supplement: Supplementary file 1 [file nanomaterials-10-01364-s001.zip › Movie S1 DEP oscillation r1.gif]

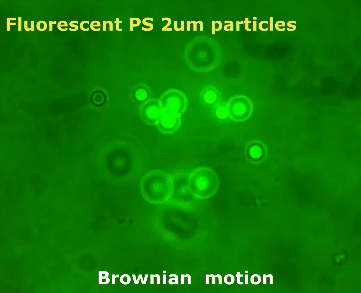

Supplement: Supplementary file 1 [file nanomaterials-10-01364-s001.zip › Movie S2 Brownie Motion and OT r1.gif]

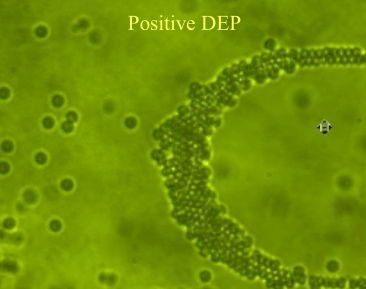

Supplement: Supplementary file 1 [file nanomaterials-10-01364-s001.zip › Movie S3 from pDEP to nDEP r1.gif]

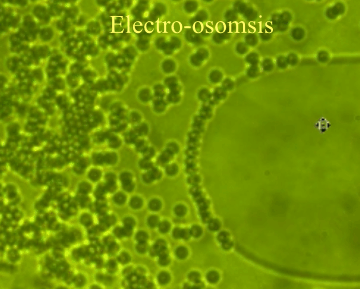

Supplement: Supplementary file 1 [file nanomaterials-10-01364-s001.zip › Movie S4 ACEO r1.gif]
